# Platypnea-Orthodeoxia Syndrome Due to Venovenous Malformation

**DOI:** 10.5935/abc.20160059

**Published:** 2016-04

**Authors:** Meng-Luen Lee, Ing-Sh Chiu

**Affiliations:** 1 Department of Pediatrics - Division of Pediatric Cardiology - Changhua Christian Children's Hospital, Changhua - Taiwan; 2 Department of Surgery - Division of Cardiovascular Surgery - Changhua Christian Children's Hospital, Changhua - Taiwan; 3 Department of Surgery - Division of Cardiovascular Surgery - College of Medicine - National Taiwan University Hospital, Taipei - Taiwan

**Keywords:** Dyspnea / etiology, Arteriovenous Malformations / etiology, Arteriovenous Malformations / surgery, Pulmonary Circulation, Septal Occluder Device

## Introduction

The platypnea-orthodeoxia syndrome (POS) is a rare clinical condition characterized by dyspnea occurring in the upright position and relieved in the supine position (platypnea) associated with oxygen desaturation noted in the upright position (orthodeoxia).^1^ A diagnosis of POS requires two of the following criteria: 1) orthodeoxia, 2) platypnea, 3) presence of an interatrial communication, 4) right-to-left shunt, and 5) absence of pulmonary arterial hypertension or right atrial hypertension.^1,2^ POS can originate in the heart, lungs, abdomen, or elsewhere due to either an intracardiac (cardiac POS) or intrapulmonary shunt, or a ventilation-perfusion mismatch.^1^ However, a venovenous malformation (VVM) as a cause of cardiac POS has never been reported in the literature.^1^

We report here the case of a 24-year-old female patient who presented with cardiac POS due to a VVM. The syndrome emerged insidiously 16 years after an extracardiac total cavopulmonary connection (TCPC) for a complex of congenital cardiovascular malformations and was successfully treated with percutaneous transvenous coil embolization.

## Case Report

A 24-year-old female patient complained of frequent yawning over the past month while standing up at work. Her lips were occasionally cyanotic, as noticed by her mother. The patient had undergone extracardiac TCPC at the age of 8 years due to a complex of congenital cardiovascular malformations, including heterotaxy/asplenia/right atrial isomerism, bilateral superior vena cava, common atrium, common atrioventricular valve, double-inlet and double-outlet left ventricle, dextro-transposition of the great arteries, supravalvular pulmonary stenosis, left aortic arch, and total anomalous pulmonary venous connection.

During evaluation at the outpatient clinic, the patient presented orthodeoxia detected by pulse oximetry, with an oxygen saturation (SpO_2_) of 96% in the supine position and 88% in the upright position. Platypnea was not observed at that time. With a diagnostic suspicion of secondary pulmonary arteriovenous malformation (PAVM) following the TCPC, the patient was admitted for further evaluation and treatment.

On admission, she weighed 63 kg and her height was 166 cm. Her blood pressure was 120/71 mmHg, pulse rate was 88 beats/min, and respiratory rate was 20 breaths/min. Laboratory tests showed a hemoglobin level of 15.6 g/dL, and normal liver function and blood coagulation tests. No information regarding the underlying cause of the orthodeoxia could be obtained from plain chest radiograph and electrocardiogram evaluations.

**Figure 1:**
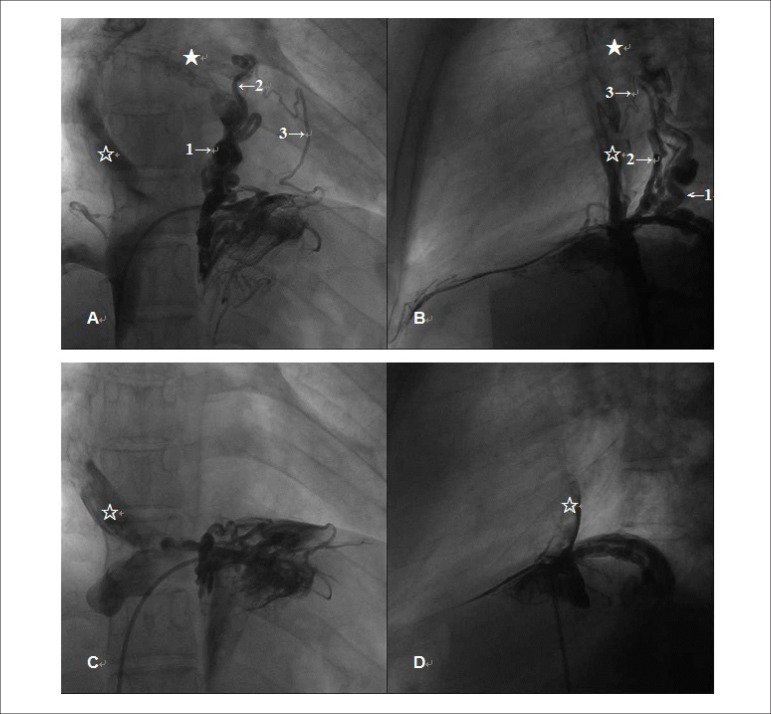
A new cardiac platypnea-orthodeoxia syndrome due to a venovenous malformation (1A and 1B). Angiography showing three anomalous systemic venous channels (venovenous malformations) draining in sequence from the inferior vena cava, hepatic vein, pulmonary veins, and pulmonary venous atrium (functional left atrium). The numbers with arrows point to the venovenous malformations, the empty star shows the total cavopulmonary connection, and the solid star shows the pulmonary venous atrium (1C and 1D). Angiography of the inferior vena cava after percutaneous transvenous coil embolization shows complete obliteration of the venovenous malformation.

Cardiac catheterization, performed after the patient granted informed consent, showed an SpO_2_ on the ascending aorta of 93%. Peak systolic pressure in the inferior vena cava, TCPC conduit, left superior vena cava, and branch pulmonary arteries were all 16 mmHg. Pulmonary capillary wedge pressure was 13 mmHg, end-diastolic left ventricular pressure was 9 mmHg, and transpulmonary capillary gradient was 7 mmHg. An angiography showed three anomalous systemic veins corresponding to a VVM draining in sequence from the inferior vena cava, hepatic vein, pulmonary veins, and pulmonary venous atrium (functional left atrium) (Figures 1A and 1B). This VVM caused a right-to-left shunt, accounting for the oxygen desaturation.

A percutaneous transvenous coil occlusion was performed uneventfully. After the procedure, an angiography of the inferior vena cava showed complete occlusion of the VVM (Figures 1C and 1D). At a 12-month follow-up, the patient reported that the yawning-orthodeoxia had disappeared, and a postural change test showed no oxygen desaturation in the upright and supine positions, with SpO_2_ measurements of ~92-96% (> 90%).

## Discussion

Table 1 summarizes the definition, mechanisms and etiologies, and diagnostic criteria of POS.

**Table 1:** Platypnea-orthodeoxia syndrome

Platypnea-orthodeoxia syndrome		
Definition^1,2^	Platypnea: dyspnea noted in the upright position and relieved in the supine positionOrthodeoxia: oxygen desaturation noted in the upright position	
Criteria*^1,2^	[1] IAC, [2] RA–LA shunt, [3] No PAH/RAH, [4] Platypnea, [5] Orthodeoxia	
Mechanisms^1,2^	[1] Intracardiac RA–LA shunt (PFO, ASD, ASA)	
	a. RA–LA shunt without pressure gradient	
	– Etiologies:	Aortic aneurysm, cardiac tamponade, pericardial effusion, constrictive pericarditis, pneumonectomy, decreased RAcompliance due to acute myocardial infarction, prominent Eustachian valve or large Chiari network, kyphosis, RA lipomatosis, RA myxoma, etc.
	b. RA–LA shunt with pressure gradient	
	– Etiologies:	PTE, idiopathic PAH, right hydrothorax, PAH in CLD, pneumonectomy, etc.
	[2] Intrapulmonary right-to-left shunt	
	– Etiologies:	PAVM, HPS, Osler-Weber-Rendu syndrome, etc.
	[3] Ventilation/perfusion mismatch	
	– Etiologies:	Emphysema, COPD, amiodarone toxicity, autonomic dysfunction, HPS, etc.

ASA:Atrial septal aneurysm; ASD: Atrial septal defect; CLD: Chronic lung disease; COPD: Chronic obstructive pulmonary disease; HPS: Hepatopulmonary syndrome; IAC: Interatrial communication; LA: Left atrial; PAH: Pulmonary arterial hypertension; PAVM: Pulmonary arteriovenous malformation; PFO: Patent foramen ovale; PTE: Pulmonary thromboembolism; RA: Right atrial; RAH: Right atrial hypertension.

* Two of these five criteria establish the diagnosis of platypnea-orthodeoxia syndrome^1,2^.

Six implications should be highlighted in the present case.

First, the patient's manifestations presented insidiously with yawning-orthodeoxia 16 years after a TCPC, thus angiography was indicated to identify the underlying causes of oxygen desaturation, which included obstruction/stenosis of the TCPC or pulmonary arteries, pulmonary arterial hypertension related to increased pulmonary arterial resistance, thrombosis of the superior vena cava, and development of a PAVM or VVM.

Second, our patient did not experience platypnea in the past or when she sought help for the problem discussed in this case. Considering the high incidence of PAVM and interatrial shunts after the Fontan operation^3^ (43% and 57%, respectively), POS is not an uncommon complication. In fact, POS emerged as a complication in eight patients with PAVM/intra-atrial shunts who underwent the Fontan operation, but none had a VVM.^3^ One of these patients had platypnea, and three others had orthodeoxia,^3^ which shows that not all patients present with platypnea and orthodeoxia simultaneously, and their detection is only possible with a postural change test.^3^ Platypnea may be difficult to identify without a careful evaluation of dyspnea,^3^ whereas orthodeoxia could be masked by profound cyanosis or even overlooked due to subtle desaturation in the upright position, as seen in our patient who had an SpO_2_ of 88%.

Patients with POS occasionally only complain of fatigue^4^ and not platypnea. In addition, assessment of our patient with the New York Heart Association (NYHA) functional classification was limited due to a mild oxygen desaturation in the upright position (orthodeoxia). Thus, semiology and oximetry were fundamental in the initial evaluation of this patient, who had presented with upright yawning and orthodeoxia. We initially considered that the complaint of yawning could be simply a subtle symptom of fatigue. However, the yawning-orthodeoxia was a manifestation of POS, which was later identified to be due to a VVM as the underlying cardiovascular pathology.

Third, VVM may cause oxygen desaturation in several cyanotic congenital heart diseases after bidirectional Glenn shunt (BDGS), with an incidence of 31-33%.^5,6^ The presence of PAVM should not preclude angiographic evaluation of the venae cavae for the establishment of the diagnosis of the VVM. It is difficult to detect a VVM before the Fontan operation,^7^ and the malformation may only become hemodynamically important after this procedure.^5-7^ Venae cavae angiography with a balloon occlusion test in the distal collector of VVM can offer a better visualization of a VVM.^7^

Fourth, a rule establishes that two clinical conditions with an anatomical and a functional component must coexist to cause POS.^1,2^ However, this rule has exceptions. Kumar et al.^8^ reported the case of a 57-year-old woman with hereditary hemorrhagic telangiectasia and PAVM who presented with POS without an interatrial defect. It holds truth for the present patient who manifested VVM as a new functional component of cardiac POS, which has never been reported in the English literature.^1,2^

Fifth, the event that orchestrated the occurrence of orthodeoxia in our patient is fascinating from an embryologic point of view. The VVM leading to the orthodeoxia was caused by a persistent communication between the proximal segment of the left umbilicovitelline vein with the left horn of the sinus venosus. Development of a VVM is known to occur due to reopening of systemic venous channels when the cavae are subjected to an elevated pressure that is transmitted backward in the pulmonary artery after a BDGS.^5-7^ In patients who underwent the Fontan operation, a positive pressure gradient between the cavae or the pulmonary arteries and the pulmonary veins (which normally ranges from 5-10 mmHg) facilitates an antegrade blood flow from the pulmonary veins to the lung.^7^ In our patient, the transpulmonary capillary gradient was 7 mmHg. Ironically, this gradient was high enough to divert the flow from the inferior cava to the anomalous left pulmonary veins, simulating a right-to-left shunt through the VVM. In addition to the influence of this gradient, gravity may have potentially increased the reversed flow from the pulmonary artery to the anomalous left pulmonary veins.^9^ Under these scenarios, a combination of decreased blood flow to the lung and increased right-to-left shunt through the VVM in our patient may have predisposed to oxygen desaturation, even if insidious. This means that in the long run, a VVM may predispose some patients with univentricular heart repaired with TCPC to present orthodeoxia or platypnea.

Sixth, a percutaneous transvenous coil occlusion is an adequate procedure to treat a small VVM.^5,6^ Guérin et al.^7^ have reported two patients with large intrahepatic VVMs causing oxygen desaturation after a modified Fontan operation. The VVMs in both patients were successfully occluded with the Amplatzer septal occluder.

## Conclusion

Both semiology and oximetry evaluation were fundamental in the initial assessment of our patient, who presented with upright yawning and mild orthodeoxia. The yawning-orthodeoxia syndrome may be a variant of POS. VVM has never been reported as a functional component of cardiac POS in the literature. Small VVM may be effectively treated by transvenous coil occlusion.
